# Clinical indicators and coronary angiographic features of expansive arterial remodelling in patients with abdominal aortic aneurysms

**DOI:** 10.1371/journal.pone.0219730

**Published:** 2019-07-16

**Authors:** Hirohisa Endo, Tomotaka Dohi, Shizuyuki Dohi, Hideki Wada, Shinichiro Doi, Yoshiteru Kato, Iwao Okai, Hiroshi Iwata, Shinya Okazaki, Kikuo Isoda, Taira Yamamoto, Katsumi Miyauchi, Atsushi Amano, Hiroyuki Daida

**Affiliations:** 1 Department of Cardiovascular Medicine, Juntendo University Graduate School of Medicine, Hongo, Bunkyo-ku, Tokyo, Japan; 2 Department of Cardiovascular Surgery, Juntendo University Graduate School of Medicine, Hongo, Bunkyo-ku, Tokyo, Japan; The Second Affilated Hospital, Zhejiang University School of Medicine, CHINA

## Abstract

The co-existence of expansive arterial remodelling in both coronary arteries (CAs) and the abdominal aorta has already been reported, although the clinical indicators and quantitative analysis have not been well studied. We therefore aimed to clarify the clinical and anatomical characteristics of patients with abdominal aortic aneurysms (AAAs). 123 AAA patients who underwent coronary angiography were compared to 123 control patients selected by propensity score matching. CA diameters of all 3 vessels were measured by quantitative coronary angiographic analysis. Coronary artery ectasia (CAE) was defined as local or generalized aneurysmal change of the CAs. Excessive expansive CA remodelling was defined as the maximal diameter of the right or left circumflex artery in the upper 75th percentile (>4.8 mm). Multivariable logistic regression analyses were used to determine predictors of CAE and excessive expansive CA remodelling. The prevalences of CAE and excessive expansive CA remodelling were significantly higher in the AAA group than in the non-AAA group (28% vs. 8% and 31% vs. 19%; both *p*<0.05). On multivariable analysis, the presence of AAA (odds ratio (OR), 4.56; 95% confidence intervals (95%CI) 2.18–10.4) and body mass index (BMI) (OR, 1.11; 95%CI 1.03–1.21) were independently associated with CAE, and higher high-sensitivity C-reactive protein (OR, 2.19; 95%CI 1.08–4.52) and BMI (OR, 1.11; 95%CI 1.02–1.21) were independently associated with excessive expansive CA remodelling. In conclusions, this study showed a higher prevalence of ectatic CA disease in AAA patients and suggests that higher inflammation and obesity are associated with expansive arterial remodelling in coronary arteries.

## Introduction

The burden of atherosclerotic cardiovascular disease is growing globally, and coronary artery disease is projected to be the leading cause of morbidity and mortality worldwide in the next decades.[1] Compensatory expansive remodelling is an important component in atherosclerosis aimed at delaying the development of significant lumen compromise. However, in arterial segments with overcompensation, this initially favourable remodelling may eventually increase the vulnerability of macrophage-rich atherosclerotic plaques.[2] Certain plaques, as a result of a phenomenon of so-called ‘arterial remodelling’ do not reduce their luminal size, presumably because of expansion of the media and external elastic membrane.[3] Although this finding may also be operative in the case of ectasia or aneurysm of various vessels, the associations of arterial remodelling between coronary arteries and the abdominal aorta are not well understood, especially in clinical practice.

The terms of aneurysmal coronary artery disease, dilating atherosclerosis or coronary artery ectasia (CAE), are commonly used to denote a more generalized defect affecting the coronary tree, often in the presence of atherosclerosis.[4, 5] CAE is a rare, yet well-recognized abnormality of the coronary anatomy. The prevalence of CAE was in the range of 1–5% of patients with suspected coronary artery disease who underwent coronary angiography (CAG). [6–8] CAE has been seen more frequently in patients with abdominal aortic aneurysms (AAAs).[9] However, there are few reports of CAE evaluated by quantitative methods, because the definition of “normal segment” has not been well established. In addition, the main mechanism underlying CAE in vascular and non-vascular pathophysiological entities is not fully identified.[10] Therefore, the aim was to investigate coronary anatomical characteristics using quantitative coronary angiography (QCA) and the clinical characteristics of CAE and coronary arterial remodelling in patients with AAAs.

## Materials and methods

### Study ethics

Written, informed consent was obtained from all patients prior to CAG. This study proceeded in accordance with the Declaration of Helsinki and with approval from Juntendo University Hospital Institutional Review Board, the ethics application approval number was 17–206.

### Study population and data collection

**Fig 1:**
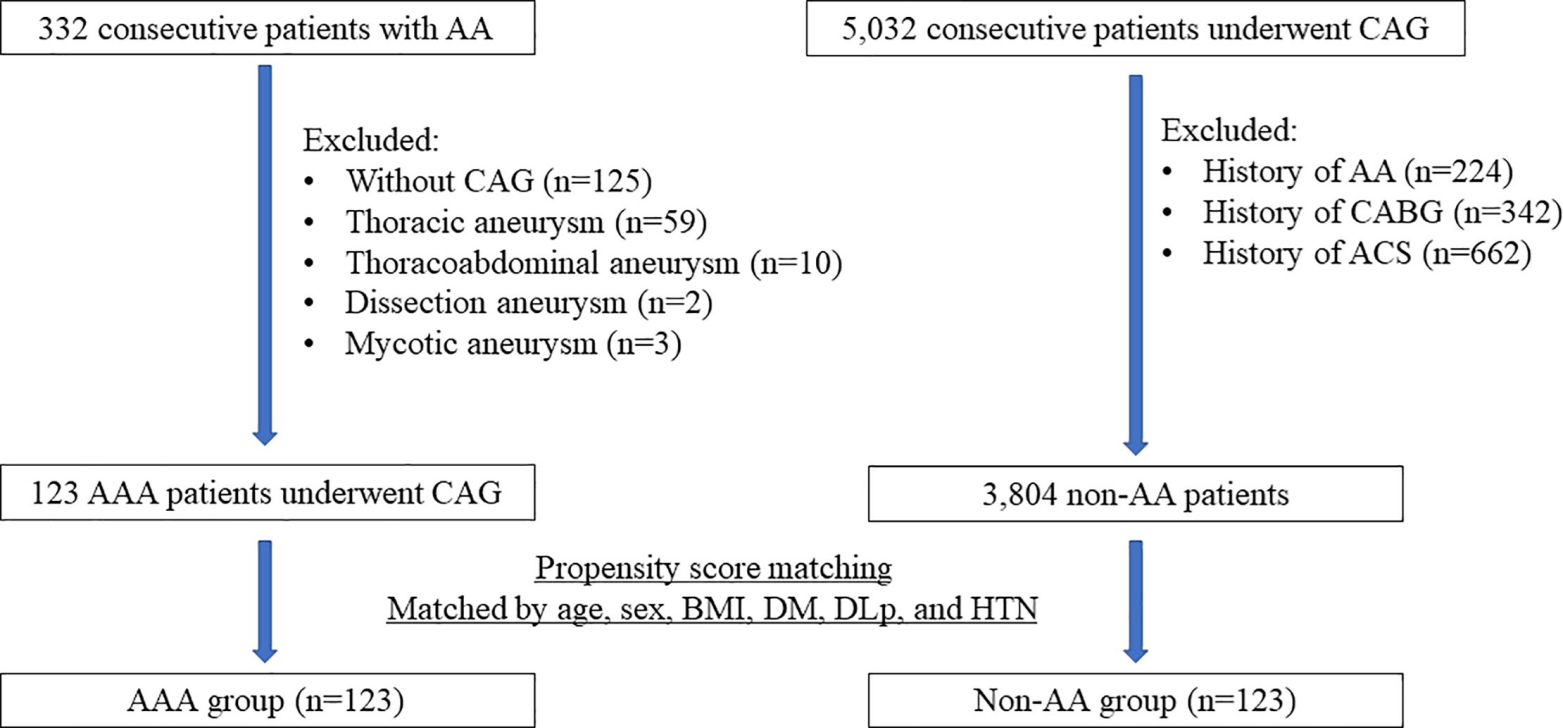
Study flow of this study. Abbreviations: AA, aortic aneurysm; AAA, abdominal aortic aneurysm; ACS, acute coronary syndrome; BMI, body mass index; CABG, coronary artery bypass graft; CAG, coronary angiography; DLp, dyslipidemia; DM, diabetes mellitus.

Three hundred thirty-two consecutive patients from an aortic aneurysmal database at the Juntendo University Hospital from January 2014 to July 2017 who underwent surgical treatment or endovascular therapy were retrospectively identified. We excluded the patients without pre-procedural CAG (n = 125), those with thoracic (n = 59), thoracoabdominal (n = 10), dissection (n = 2), mycotic aneurysm (n = 3), and the patients who did not obtain adequate angiographic images for QCA analysis because of poor filling of the vessels with contrast (n = 10). A total of 123 patients with angiographically evaluated coronary arterial anatomy and diameter who had an AAA constituted the AAA group. During the same period, a control group was selected from 5,603 consecutive patients who underwent CAG in our institution. From this CAG database, patients with a history of aortic aneurysm, acute coronary syndrome, and coronary artery bypass graft were excluded. To minimize confounding variables related to coronary risk factors, propensity score matching was used to derive the risk factor-matched controls as the non-AAA group. The flow chart of this study was presented Fig 1.

Demographic data, coronary risk factors, and medication use at the time of CAG were collected from our institutional medical records. All blood samples were obtained before elective CAG.

### Angiographic measurements and definition

Coronary angiography was routinely performed by experienced interventional cardiologists in multiple projections. Two independent analysts used validated software (QAngio XA, Medis, Leiden, The Netherlands). Before analysis, the analysts selected a frame that was filled with contrast medium, shown as a sufficiently clear image to analyse the target segment, that did not overlap with branches of the coronary artery. Furthermore, the automated contour detection was affected by image quality, especially in ectatic coronary arteries. Therefore, manual correction of the vessel border was often necessary with this software.

The right dominant coronary artery and the left dominant coronary artery are the posterior descending arteries originating from the right coronary artery (RCA) and left circumflex (LCX), respectively. In the case of a balanced coronary system, the RCA supplied the posterior descending artery, and the LCX gave rise to a posterolateral artery with an occasional additional parallel posterior descending branch.

CAE was defined as abnormal dilatation of the epicardial coronary arterial segment to a diameter ≥150% of the adjacent healthy normal artery or normal segment of the same vessel.[5] If no adjacent normal segment could be identified, the diameter of the largest healthy coronary artery of the patient was considered the reference normal value. This approach included the case when the entire artery was conspicuously large without a normal segment.[11] The severity of CAE was determined according to the Markis classification.[8] Diffuse ectasia of 2 or 3 vessels was classified as type I, diffuse disease in 1 vessel and localized disease in another vessel as type II, diffuse ectasia of only 1 vessel as type III, and localized segmental ectasia as type IV.

**Fig 2:**
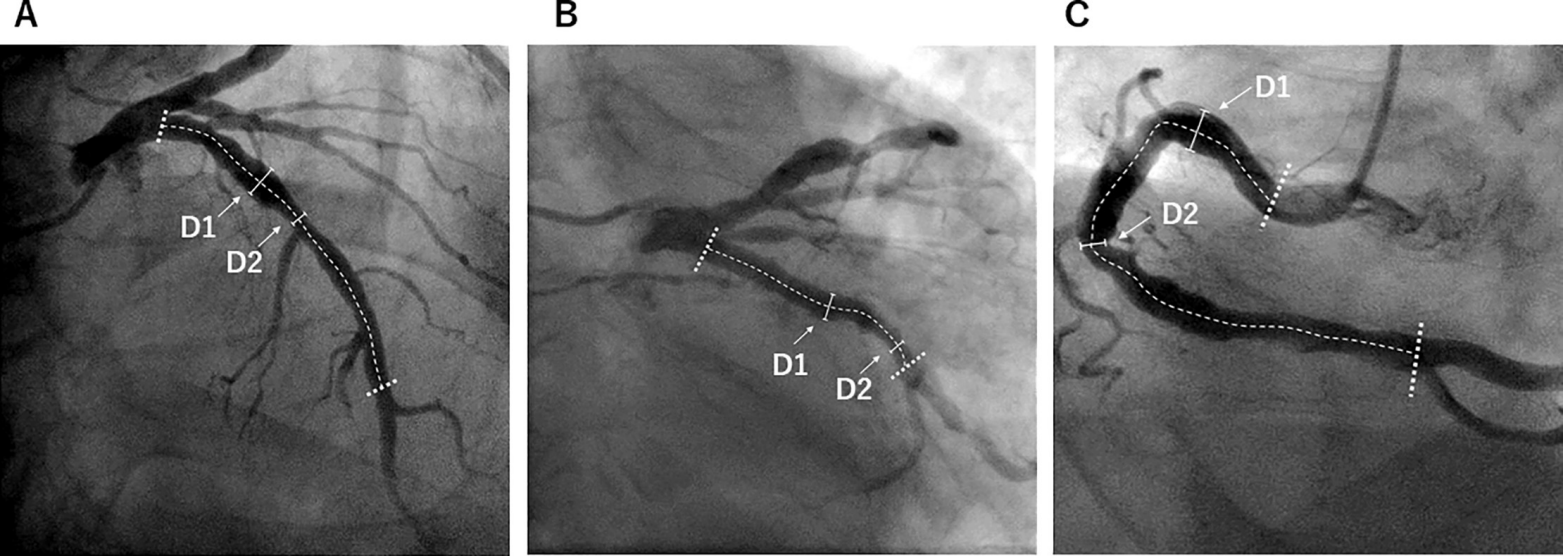
The parameter assessment of ectatic coronary arteries. The dotted lines show the range analysed in the left anterior descending artery (A), left circumflex artery (B), and right coronary artery (C), respectively. Abbreviations: D1, maximal diameter; D2, minimal diameter.

In the present study, the mean, minimal, and maximal coronary diameters were measured as topographical parameters in all 3 coronary vessels of each patient by QCA analyses. The anatomical landmarks for QCA analyses were defined as follows: (1) in the RCA, the proximal landmark was the ostium of the RCA, and the distal landmark was the first branch arising from the posterior descending artery; (2) in the LCX, the proximal landmark was the origin of the LCX, and the distal landmark was the distal-major branch arising from the posterior lateral artery; and, (3) in the left anterior descending artery (LAD), the proximal landmark was the origin of the LAD, and the distal landmark was the midpoint between the first major septal branch and the apex (the end of segment 7 according to the American College of Cardiology and the American Heart Association classification of coronary artery segments). Excessive expansive coronary arterial (CA) remodelling, defined as the maximal diameter of the RCA or LCX considering coronary artery dominance greater than the upper 75th percentile value, was estimated for further statistical analyses. The coronary artery diameter index was defined as the maximal diameter divided by the body surface area (BSA) of the patient. The basic parameter assessment of ectatic coronary arteries is shown in Fig 2.

### Statistical analysis

Continuous variables are expressed as means ± standard deviation or medians (interquartile range; IQR) depending on their distribution. Categorical variables are expressed as percentages. Continuous variables were compared using Student’s *t*-test or the Wilcoxon rank-sum test. Categorical variables were compared using the chi-squared test. Propensity scores were calculated, using age, sex, body mass index (BMI), and the presence of diabetes mellitus, dyslipidaemia, and hypertension as covariates. Patients in the two groups were matched through the greedy matching protocol with a fixed calliper width of 0.20.

Logistic regression analyses were used to estimate the relative risk of CAE and of excessive expansive CA remodelling, respectively. Model 1 was adjusted for variables showing values of p < 0.05 on univariate analyses (CAE: AAA and BMI, Excessive expansive CA remodelling: AAA, BMI, LDL-C, HDL-C, and higher high-sensitivity C-reactive protein (hs-CRP)). Model 2 was adjusted for the variables in model 1 plus age and current smoking. Odds ratios (ORs) and 95% confidence intervals (CIs) were calculated. A higher hs-CRP was defined as hs-CRP > 0.1 mg/dL according to the optimal cut-off value of the receiver operating characteristic curve analysis. A *p* value < 0.05 was considered significant. All data were analysed using JMP version 12.2 for Windows (SAS Institute, Cary, NC, USA).

## Results

### Clinical characteristics and laboratory data

A total of 123 patients with AAA and 123 patients without AAA were identified and included in the analysis. Table 1 shows the patients’ clinical characteristics and laboratory data. Overall, the patients’ mean age was 72 years, and 85% of patients were male. The prevalences of hypertension, diabetes, and dyslipidaemia were similar between the two groups. In the AAA group, abdominal aortic aneurysm diameter was 50.6 ± 9.7 mm. On the other hand, the maximum abdominal aortic diameter in the non-AAA patients was 20.3 ± 4.8 mm. There was no significant difference in medication use between the two groups except for aspirin intake.

**Table 1:** Baseline clinical characteristics, laboratory, and medication data of the study population.

	Total	AAA group	Non-AAA group	*p* value
**Patients’ characteristics**	n = 246	n = 123	n = 123	
Age, y	72 ± 9	73 ± 9	72 ± 10	0.91
Male, n (%)	210 (85)	104 (85)	106 (86)	0.72
BMI, kg/m^2^	24.3 ± 4.0	24.2 ± 4.1	24.3 ± 4.0	0.84
Hypertension, n (%)	210 (85)	106 (86)	104 (85)	0.72
Diabetes mellitus, n (%)	75 (31)	33 (27)	42 (34)	0.21
Dyslipidaemia, n (%)	211 (86)	103 (84)	108 (88)	0.36
Current smoking	47 (19)	29 (24)	18 (15)	0.07
Aneurysmal maximal diameter, mm	38 ± 17	51 ± 10	20 ± 5	<0.001
**Laboratory data**				
TC, mg/dL	183 ± 39	184 ± 36	182 ± 41	0.58
LDL-C, mg/dL	109 ± 33	109 ± 32	108 ± 34	0.87
HDL-C, mg/dL	47 ± 14	47 ± 13	47 ± 15	0.98
TG, mg/dL	117 (85, 165)	123 (87, 168)	114 (83, 162)	0.22
Lp(a), mg/dL	17 (8, 33)	18 (9, 33)	15 (8, 31)	0.12
HbA1c, %	6.1 ± 0.8	6.1 ± 0.8	6.1 ± 0.7	0.76
eGFR, ml/min/1.73 m^2^	66 ± 24	65 ± 23	68 ± 25	0.37
hs-CRP, mg/dL	0.09 (0.05, 0.19)	0.11 (0.05, 0.27)	0.08 (0.03, 0.18)	0.02
NLR	2.2 (1.5, 2.9)	2.4 (1.9, 3.2)	1.8 (1.4, 2.6)	<0.001
Adiponectin, μg/mL	8.8 (5.9, 14.7)	9.4 (5.5, 15.4)	8.4 (5.9, 14.4)	0.81
MMP-3, ng/mL	57 (43, 81)	60 (43, 82)	53 (42, 82)	0.35
MMP-9, ng/mL	28 (19, 48)	28 (18, 45)	30 (20, 53)	0.14
**Medication data**				
Aspirin, n (%)	87 (35)	36 (29)	51 (42)	0.045
Statins, n (%)	135 (55)	68 (55)	67 (55)	0.90
CCBs, n (%)	120 (49)	60 (49)	60 (49)	1.00
ACE-Is/ARBs, n (%)	118 (56)	45 (51)	73 (59)	0.20
Beta-blockers, n (%)	79 (32)	37 (30)	42 (34)	0.49
Insulin, n (%)	12 (5)	5 (4)	7 (6)	0.55

AAA, abdominal aortic aneurysm; ACE-I, angiotensin-converting enzyme inhibitor; ARB, angiotensin receptor blocker; BMI, body mass index; CCB, calcium channel blocker; eGFR, estimated glomerular filtration rate; HbA1c, haemoglobin A1c; HDL-C, high-density lipoprotein-cholesterol; hs-CRP, high-sensitivity C-reactive protein; LDL-C, low-density lipoprotein-cholesterol; Lp(a), lipoprotein(a); MMP, matrix metalloproteinase; NLR, neutrophil-to-lymphocyte ratio; TC, total cholesterol; TG, triglycerides.

The haemoglobin A1c level, estimated glomerular filtration rate, and lipid profiles including low density lipoprotein-cholesterol (LDL-C), high density lipoprotein-cholesterol (HDL-C), triglycerides, and lipoprotein (a) levels were similar in the two groups. In contrast, inflammatory markers such as the neutrophil-to-lymphocyte ratio (NLR) and hs-CRP levels were significantly higher in the AAA group than in the non-AAA group (NLR: 2.4 (IQR: 1.9–3.2) vs. 1.8 (IQR: 1.4–2.6), *p*<0.001; hs-CRP: 0.11 mg/dL (IQR: 0.05–0.27 mg/dL) vs. 0.08 mg/dL (IQR: 0.03–0.18 mg/dL), *p* = 0.02). Serum adiponectin, matrix metalloproteinase (MMP)-3, and plasma MMP-9 levels were not significantly different between the AAA group and the non-AA group.

### Coronary angiographic characteristics

**Fig 3:**
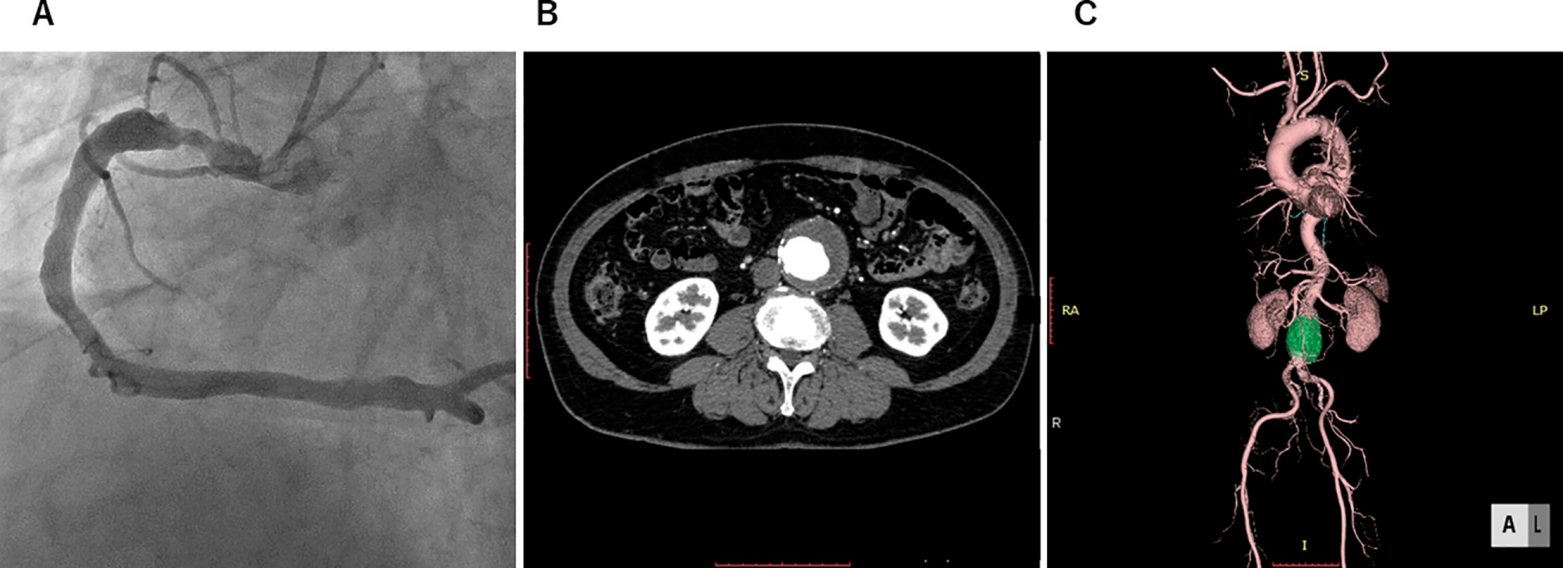
A representative case of coronary artery ectasia. Images of a 62-year-old man with an abdominal aortic aneurysm. Diffuse ectasia and focal stenosis in the right coronary artery are seen (A). The axial image (B) and 3D reconstruction image (C) of computed tomography show an infra-renal aortic aneurysm.

The overall prevalence of CAE was estimated as 18%; it was significantly higher in the AAA group (28%) than in the non-AAA group (8%) (*p*<0.001). Table 2 shows the angiographic characteristics of CAE. Ectatic involvement was more frequent in the RCA (80%) compared to the LAD (41%) and LCX (32%). In patients with CAE, most cases (77%) were classified as diffuse. Using the Markis classification of CAE, type III ectasia was the most common (39%), followed by type IV (25%), type I (21%), and type II (16%). Patients with CAE had a higher BMI than those without CAE (BMI: 25.6 ± 0.6 kg/m^2^ vs. 24.0 ± 0.3 kg/m^2^, *p* = 0.01). There were no significant differences in age, sex, prevalences of hypertension, diabetes, dyslipidaemia, and chronic kidney disease, and smoking status between the patients with and without CAE. A representative case is shown in Fig 3.

**Table 2:** Angiographic characteristics of coronary artery ectasia.

**CAE prevalence**	44/246 (18%)
** **AAA/Non-AAA	34/10
**Coronary artery dominance, n (%)**	
** **RCA	39 (89)
** **LCX	3 (7)
** **Balanced	2 (5)
**Multivessel disease, n (%)**	15 (34)
**Markis classification, n (%)**	
** **Class I	9 (21)
** **Class II	7 (16)
** **Class III	17 (39)
** **Class IV	11 (25)
**Ectatic vessel, n (%)**	
** **LAD	18 (41)
** **LCX	14 (32)
** **RCA	35 (80)

AAA, abdominal aortic aneurysm; CAE, coronary artery ectasia; LAD, left anterior descending artery; LCX, left circumflex; RCA, right coronary artery.

The results of QCA analysis are shown in Table 3. Right coronary artery dominance was found in 97/123 cases (79%) of the AAA group and 84/123 cases (68%) of the non-AAA group (*p* = 0.02). The maximal diameters in all 3 coronary arteries were significantly larger in the AAA group than in the non-AAA group (LAD: 4.0 ± 0.7 mm vs. 3.4 ± 0.6 mm; LCX: 3.8 ± 0.9 mm vs. 3.5 ± 0.8 mm; RCA: 4.4 ± 1.3 mm vs. 4.0 ± 0.9 mm, all *p* < 0.05). Even considering coronary artery dominance or BSA, coronary arterial diameter was larger in the AAA patients than in the non-AAA patients. The prevalence of excessive expansive CA remodelling (>4.8 mm) was significantly higher in the AAA group (31%) than in the non-AAA group (19%) (*p* = 0.03).

**Table 3:** Qualitative and quantitative coronary angiography findings.

	Total	AAA group	Non-AAA group	*p* value
	n = 246	n = 123	n = 123	
**Prevalence of CAE, n (%)**	44 (18)	34 (28)	10 (8)	<0.001
**Maximal diameter, (mm)**				
** **LAD	3.69 ± 0.72	3.95 ± 0.71	3.43 ± 0.63	<0.001
** **LCX	3.64 ± 0.84	3.81 ± 0.85	3.48 ± 0.80	0.003
** **RCA	4.20 ± 1.10	4.37 ± 1.29	4.02 ± 0.85	0.01
** **Maximal diameter of RCA or LCX	4.44 ± 0.88	4.62 ± 0.98	4.26 ± 0.72	0.001
**Excessive expansive CA remodelling, n (%)**	61 (25)	38 (31)	23 (19)	0.03
**CA diameter index, (mm/m**^**2**^**)**				
** **LAD	2.19 ± 0.43	2.33 ± 0.41	2.04 ± 0.41	<0.001
** **LCX	2.16 ± 0.54	2.25 ± 0.56	2.07 ± 0.51	0.01
** **RCA	2.48 ± 0.64	2.58 ± 0.76	2.39 ± 0.49	0.02
**Mean diameter, (mm)**				
** **LAD	2.64 ± 0.50	2.84 ± 0.47	2.44 ± 0.45	<0.001
** **LCX	2.74 ± 0.67	2.89 ± 0.68	2.59 ± 0.63	<0.001
** **RCA	3.08 ± 0.73	3.20 ± 0.83	2.96 ± 0.61	0.01
**Minimal diameter, (mm)**				
** **LAD	1.66 ± 0.60	1.86 ± 0.62	1.46 ± 0.50	<0.001
** **LCX	1.79 ± 0.69	1.96 ± 0 .73	1.62 ± 0.61	<0.001
** **RCA	1.97 ± 0.79	2.13 ± 0.82	1.81 ± 0.73	0.002

Excessive expansive CA remodelling defined as the maximal diameter of the RCA or LCX considering coronary artery dominance greater than the upper 75th percentile value (4.8 mm). CA diameter index defined as the maximal diameter divided by the body surface area.

In patients with the AAA and non-AAA group, correlations between the maximal diameter of the RCA or LCX and age (*r* = -0.16, *p* = 0.01), BMI (*r* = 0.22, *p*<0.001), LDL-C (*r* = 0.18, *p*<0.01), and log-transformed hs-CRP (*r* = 0.18, *p*<0.01) were significant, but relatively weak.

### Predictors of coronary artery ectasia and expansive coronary arterial remodelling

Table 4 summarizes the results of multivariate logistic regression analyses for CAE and excessive expansive CA remodelling. On multivariate analysis, the presence of AAA and BMI were independently associated with CAE: presence of AAA (adjusted OR, 4.56; 95%CI, 2.18–10.4; *p* < 0.001) and BMI (per kg/m^2^; adjusted OR, 1.11; 95%CI, 1.03–1.21; *p* = 0.01). Meanwhile, significant predictors for excessive expansive CA remodelling were BMI (per kg/m^2^; adjusted OR, 1.11; 95%CI, 1.02–1.21; *p* = 0.02) and higher hs-CRP (adjusted OR, 2.19; 95%CI, 1.08–4.52; *p* = 0.03).

**Table 4:** Multivariable logistic regression models for coronary artery ectasia and excessive expansive coronary artery remodelling.

	OR	95%CI	p value
**Model 1**^a^			
**CAE**			
** **AAA	4.56	2.18–10.4	<0.001
** **BMI, 1 kg/m^2^ increase	1.11	1.03–1.21	0.01
**Dilated coronary arterial remodeling**			
** **AAA	1.67	0.83–3.39	0.15
** **BMI, 1 kg/m^2^ increase	1.11	1.02–1.21	0.02
** **LDL-C, 1 mg/dL increase	1.01	0.99–1.02	0.17
** **HDL-C, 1 mg/dL increase	0.98	0.95–1.01	0.07
** **HS-CRP > 0.1 mg/dL	2.19	1.08–4.52	0.03
**Model 2**^b^			
**CAE**			
** **AAA	4.77	2.27–10.9	<0.001
** **BMI, 1 kg/m2 increase	1.11	1.02–1.22	0.02
** **Age, 1-year increase	0.99	0.96–1.04	0.86
** **Current smoking	0.61	0.23–1.49	0.29
**Dilated coronary arterial remodeling**			
** **AAA	1.70	0.84–3.48	0.14
** **BMI, 1 kg/m^2^ increase	1.08	0.98–1.19	0.11
** **LDL-C, 1 mg/dL increase	1.01	0.99–1.02	0.14
** **HDL-C, 1 mg/dL increase	0.97	0.95–1.001	0.06
** **HS-CRP > 0.1 mg/dL	2.37	1.16–4.95	0.02
** **Age, 1-year increase	0.97	0.93–1.01	0.19
** **Current smoking	0.57	0.21–1.42	0.24

Excessive expansive CA remodelling defined as the maximal diameter of the RCA or LCX considering coronary artery dominance greater than the upper 75th percentile value (4.8 mm). Abbreviations: AAA, abdominal aortic aneurysm; BMI, body mass index; CA, coronary artery; CAE, coronary artery ectasia; HDL-C, high-density lipoprotein-cholesterol; hs-CRP, high-sensitivity C-reactive protein; LDL-C, low-density lipoprotein-cholesterol; OR, odds ratio, 95%CI, 95% confidence interval.

^a^ Multivariable model 1: variables showing values of p < 0.05 on univariate analyses.

^b^ Multivariable model 2: model 1 + age and current smoking.

## Discussion

The major findings of the present study are as follows: 1) CAE was found more frequently in patients with AAA (28%) than in propensity-matched control patients without AAA (8%); 2) in the QCA analyses, the diameters of the conduit coronary arteries were consistently larger in patients with AAA than in patients without AAA; and 3) the presence of AAA was strongly associated with CAE, and higher hs-CRP and BMI were significantly associated with excessive expansive CA remodelling.

The present results include several findings concerning excessive expansive arterial remodelling in coronary arteries (muscular arteries) and the abdominal aorta (elastic artery). Although previous reports showed a prevalence rate of CAE of approximately 20% in AAA patients, a slightly high prevalence rate of CAE was observed in our results. In addition, CAE was also frequently observed in patients with ascending aorta aneurysms or bicuspid aortic valves.[11, 12] The major histological features of AAA include chronic medial and adventitial inflammation with medial degeneration, including smooth muscle cell apoptosis and excessive loss of extracellular matrix (ECM), especially extensive elastin fragmentation.[13, 14] Similarly, ectatic coronary arterial walls also demonstrate marked degradation of the medial collagen and elastin fibres, with disruption of the internal and external elastic lamina.[4] Degradation of ECM components by MMP enzymes contributes to the pathogenesis of vessel wall remodelling. A recent meta-analysis showed a significant difference in MMP-9, tissue inhibitor of matrix metalloproteinase 1 (TIMP-1), and CRP between patients with and without AAA [15]; MMP-3 and MMP-9 levels were higher in patients with CAE than in patients with coronary artery disease and normal coronary arteries.[16] In the present study, there were no significant differences in MMPs in patients with/without AAA and with/without CAE. A possible explanation is that activation of MMPs was observed not only in patients with AAA and CAE, but also in patients with collagen diseases and other chronic inflammatory disorders. Moreover, increased activity of MMPs in patients with CAE may lead to local tissue consumption and result in decreased levels in the circulation.[17, 18]

In the present study, increased BMI was an independent predictor for both CAE and excessive expansive CA remodelling. Police et al. reported that inflammation in periaortic adipose tissue surrounding abdominal aortas of angiotensin-II infused obese mice was associated with AAA formation.[19] From the Framingham Offspring and Third Generation cohort, periaortic fat volume was associated with abdominal and thoracic aortic diameters, while adipokines (resistin and adiponectin) had no significant impact on these associations.[20] Yunget al. demonstrated that there was a significant positive correlation between pericardial fat volume and CAE estimated by computed tomography.[21] A previous autopsy study reported that BMI and total epicardial fat weight were correlated in subjects with both normal hearts and those with ischemic disease.[22] Recently, in a prospective cohort study, BMI was reported to be one of the factors associated with CAE.[23] Based on these results, a higher BMI, namely obesity, was likely responsible for the process of expansive arterial remodelling.

The present study also showed that higher inflammation was significantly associated with coronary arterial remodelling. Previous reports showed that higher CRP was significantly associated with the presence of CAE and AAA.[10, 15] Johnston et al. recently reported that inhibition of interleukin-1β (IL-1β) decreased thoracic aortic aneurysm formation.[24] In addition, in a limited clinical case report presentation, a recombinant IL-1β receptor antagonist, anakinra, decreased the progression of coronary aneurysms in Kawasaki disease.[25] The present results also showed that elevated CRP levels were significantly associated with excessive expansive CA remodelling.

To the best of our knowledge, there has not been a previous study evaluating both CAE and expansive CA remodelling using a quantitative method. Comparing the AAA group to the non-AAA group, the present result shows that the presence of AAA is a strong predictor of CAE. In other words, the possible existence of AAA should be considered when we occasionally find typical CAE on coronary angiography. However, in the present analysis, the predictors of CAE and excessive expansive CA remodelling were different. One possible explanation for this finding is that excessive expansive CA remodelling may include not only typical CAE, but also positive arterial remodelling compensating for lumen narrowing.

This study had several limitations. First, this was a retrospective observational study from a patient cohort with aortic aneurysms. It was also not a pure prevalence study. Second, we selected the patients who underwent CAG before surgical or endovascular therapy for AAA. However, the decision to perform CAG was based on not only cardiovascular risk and coronary artery calcification, but also physician’s discretion. Third, excessive expansive CA remodelling defined as the upper 75th percentile value might include normal coronary arteries. However, the matching controls had many risk factors for coronary atherosclerosis. Therefore, they cannot be assessed simply as normal vessels.

In conclusion, the presence of AAAs was significantly associated with CAE. The results of the present study suggest possible associations of higher inflammation and obesity with expansive arterial remodelling. Thus, further studies are necessary to clarify the effects of adipose tissue inflammation on the vascular wall.
